# Changes in pulmonary endothelial cell properties during bleomycin-induced pulmonary fibrosis

**DOI:** 10.1186/s12931-018-0831-y

**Published:** 2018-06-26

**Authors:** Shinpei Kato, Naoki Inui, Akio Hakamata, Yuzo Suzuki, Noriyuki Enomoto, Tomoyuki Fujisawa, Yutaro Nakamura, Hiroshi Watanabe, Takafumi Suda

**Affiliations:** 1 Second Division, Department of Internal Medicine, Hamamatsu University School of Medicine, 1-20-1 Handayama, Hamamatsu, 431-3192 Japan; 2 Department of Clinical Pharmacology and Therapeutics, Hamamatsu University School of Medicine, 1-20-1 Handayama, Hamamatsu, 431-3192 Japan

**Keywords:** α-SMA, Bleomycin, Endothelial cell, Fibrosis, Nitric oxide, Prostaglandin I_2_, TGF-β

## Abstract

**Background:**

Pulmonary fibrosis is a progressive and lethal disease characterized by damage to the lung parenchyma with excess extracellular matrix deposition. The involvement of endothelial cells in fibrosis development is unclear.

**Methods:**

We isolated pulmonary endothelial cells, using a magnetic-activated cell sorting system, from mice with pulmonary fibrosis induced by intratracheal bleomycin. We characterized endothelial cells isolated at various times in the course of pulmonary fibrosis development.

**Results:**

Inflammatory cell infiltration was observed at 7 days after bleomycin administration, and fibrotic changes with increased collagen content were observed on day 21. Endothelial cells were isolated at these two timepoints. Levels of von Willebrand factor, plasminogen activator inhibitor-1 and matrix metalloproteinase-12 were elevated in lung endothelial cells isolated from bleomycin-treated mice at days 7 and 21. This indicated that intratracheal bleomycin administration induced endothelium injury. Expression of fibrogenic mediators, transforming growth factor (TGF)-β, connective tissue growth factor and platelet-derived growth factor-C was elevated in the cells from bleomycin-treated, compared with untreated, lungs. When endothelial cells were treated with TGF-β, α-smooth muscle actin (SMA) expression and collagen production were increased only in those cells from bleomycin-treated mouse lungs. Thapsigargin-induced prostaglandin I_2_ and nitric oxide production, decreased in endothelial cells from bleomycin-treated mouse lungs, compared with controls, was further suppressed by TGF-β.

**Conclusion:**

Bleomycin administration induced functional changes in lung endothelial cells, indicating potential involvement of endothelium in pulmonary fibrogenesis.

**Electronic supplementary material:**

The online version of this article (10.1186/s12931-018-0831-y) contains supplementary material, which is available to authorized users.

## Background

Interstitial lung diseases (ILDs) and pulmonary fibrosis are characterized by damage to the lung parenchyma with varying patterns of inflammation and fibrosis [1]. There are hundreds of estimated etiological factors, and the pathogenesis is not completely understood [2]. It is currently believed that epithelial cell injury initiates the pathology and is followed by a mild inflammatory response [2–4]. Subsequently, fibrosis, with excess extracellular matrix deposition and collagen accumulation, occurs as a dysregulated repair process, resulting in destruction of the lung architecture, that causes ventilatory impairment and respiratory failure [1]. Among fibrotic disorders, idiopathic pulmonary fibrosis (IPF) with its pathological features of usual interstitial pneumonia (UIP), is a chronic progressive and lethal fibrotic disease with a median survival of 2–3 years after diagnosis [2, 3, 5]. It is among the most common forms of ILDs and is widely studied in the clinical and research fields [2, 3].

The primary site of injury is the interstitium, forming the space between the epithelial and endothelial basement membranes [1]. Although fibroblasts are key fibrosis effectors [4, 6], fibrotic changes are caused by complex activities within several cell types including epithelial, inflammatory and fibrogenic effector cells [6, 7]. Pulmonary endothelial cells continuously cover the intravascular lumen and are anatomically close to epithelial and mesenchymal cells [8], suggesting that endothelium could also play an important role in the process of pulmonary fibrosis [6, 9, 10]. Takabatake et al. demonstrated pulmonary microvascular endothelial cell injury during a fibrotic process in patients with ILDs [9]. In patients with ILDs, the rate of ^123^I-metaiodobenzylguanidine washout from the lungs, reflecting pulmonary endothelial cell injury, was lower than in normal controls.

Additionally, endothelial cells may be involved in pulmonary fibrosis through two mechanisms. One is a pathway involving production of the gaseous free radical nitric oxide [11], as regulated by three isoforms of nitric oxide synthase (NOS), neuronal NOS, inducible NOS and endothelial NOS (eNOS) [12]. Nitric oxide regulates multiple biological processes and maintains respiratory homeostasis [11] but, at high levels, it can instead induce harmful inflammation and apoptosis. Several reports showed that nitric oxide is important for development of pulmonary fibrosis. Nitric oxide protects cells from oxidant-induced injury, and eNOS knockout animals have prolonged fibrosis after bleomycin exposure [13]. Yoshimura et al. showed that eNOS overexpression attenuated bleomycin-induced histological changes, lung collagen accumulation and mortality in eNOS transgenic mice [14]. They also showed that eNOS overexpression occurred mainly in endothelial cells, suggesting involvement of the endothelium, through a nitric oxide dependent mechanism, in pulmonary fibrosis development.

The other potential mechanism of endothelial cell involvement in fibrosis is their ability to transform to myofibroblasts during fibrogenesis. Myofibroblasts are defined as fibroblast-like cells expressing α-smooth muscle actin (SMA) [3, 6, 15, 16] and are among the primary effector cells in tissue remodeling and fibrosis [6, 17]. During lung injury, myofibroblast numbers increase and they produce high amounts of collagen and other extracellular matrix components during a repair and reconstruction process. Proposed origins of myofibroblasts include resident lung fibroblasts differentiating into activated myofibroblasts, differentiation of epithelial cells into mesenchymal cells and recruitment of circulating fibrocytes and bone marrow-derived progenitor stem cells [6, 17, 18]. Recently, transition of endothelial cells into a mesenchymal phenotype, known as the endothelial to mesenchymal transition (or endothelial–mesenchymal transition), was proposed to be another source of myofibroblasts [17, 18]. This transition process is defined based on endothelial and mesenchymal markers and morphology. Endothelial cells lose their specific morphology and markers and acquire mesenchymal, myofibroblast-like properties. However, functional changes occurring in endothelial cells during this phenotypic alteration remain unknown.

Although no model is completely consistent with pulmonary fibrosis in humans, bleomycin is most widely used to induce pulmonary fibrosis in experimental animals [19, 20]. After systemic delivery of bleomycin, endothelial cells were directly injured [21] and this was subsequently followed by epithelial cell injury, inflammation and fibrosis [19, 22]. With airway administration of bleomycin, airway epithelial cells were injured initially, but the development of endothelial cell damage has not been fully elucidated. The goal of our study was to characterize pulmonary endothelial cells isolated from an in vivo mouse model of pulmonary fibrosis induced by intratracheally administered bleomycin. Endothelial cells have functional and phenotypic plasticity, influenced by their surrounding microenvironments [8]. Therefore, in particular, we investigated functional changes in the endothelial cells and their production of fibrosis-related mediators, intracellular nitric oxide and prostaglandin I_2_ (PG-I_2_) during the process of bleomycin-induced lung injury.

## Methods

### Bleomycin-induced mouse pulmonary fibrosis model

Male C57/BL6 mice 8 to 12 weeks old (20–25 g, SLC, Shizuoka, Japan) were anesthetized with intraperitoneal ketamine (80 mg/kg) and xylazine (10 mg/kg) and received a single tracheal injection of 2 mg/kg bleomycin sulfate (Nippon Kayaku, Tokyo, Japan) in 50 μL sterile saline using a Microsprayer Aerosolizer (PennCentury, Philadelphia, PA, USA) on day 0. All mice were killed by cervical dislocation and lungs were harvested at day 7, 14, 21 or 28 after intratracheal bleomycin or saline treatment. This study was approved by the Animal Care and Use Committee of Hamamatsu University School of Medicine and all experiments were performed according to guidelines of this Committee.

### Bronchoalveolar lavage (BAL)

After each mouse was sacrificed, a 22-gauge catheter was inserted into the trachea and 1 mL saline was injected and collected in three consecutive washes. After centrifugation, cell pellets were suspended, and total cells were counted using a hemocytometer in the presence of Turk’s solution. Each cell suspension was smeared on a glass slide and this was stained with Diff-quick. Differential cell counting was performed manually under a light microscope. Total protein content was quantitated using a DC Protein Assay kit (Bio-Rad Laboratories, Hercules, CA, USA) [23]. Concentrations of murine transforming growth factor (TGF)-β1 in the supernatant were evaluated using ELISA kits (R&D systems, Minneapolis, MN, USA) according to the manufacturer’s protocol.

### Histopathology

The lungs were quickly removed, inflated using a syringe, fixed in 10% buffered formalin, and embedded in paraffin. Sections (4 μm) cut from embedded tissues were stained with hematoxylin-eosin (HE) and Masson’s trichrome stains and observed under a light microscope.

### Hydroxyproline assay

Collagen content in the mouse lungs was measured using a hydroxyproline assay. Lung tissue was homogenized with 100 μL distilled water per 10 mg tissue. HCl (12 M, 100 μL) was added to each homogenate in a pressure-tight glass tube and samples were hydrolyzed at 120 °C for 12 h. A colorimetric assay was performed (QuickZyme Biosciences, Leiden, Netherlands) according to the manufacturer’s protocol.

### Isolation of mouse lung endothelial cells

After harvesting, lungs were minced with sterile scissors and incubated with 200 U/mL collagenase type 2 (Worthington, Lakewood, NJ, USA) and 100 U/mL DNase 1 (Worthington) for 30 min at 37 °C in phosphate-buffered saline (PBS). Lung tissue was dissociated into single cell suspensions using a gentle MACS Dissociator (Myltenyi Biotechnology, Bergisch Gladbach, Germany), according to the manufacturer’s protocol. CD45 microbeads (Myltenyi Biotec) were incubated with the single cell suspensions, enabling negative selection by magnetic cell separation using a Midi MACS separator (Myltenyi Biotec). Then CD31 microbeads (Myltenyi Biotec) were incubated in the CD45 negative cell population and positively selected by magnetic cell separation using the same separator. The CD45^−^CD31^+^ cell population thus obtained was cultured as mouse lung endothelial cells. For RNA isolation, cells were lysed in RLT buffer (Qiagen, Valencia, CA, USA). Purity of magnetically selected CD45^−^CD31^+^ endothelial cells, confirmed by flow cytometry, was greater than 90% (Additional file 1: Fig. S1).

### Cell culture

Endothelial cells were cultured at a density of 1 × 10^6^ cells/well on 0.5% gelatin-coated black-walled and clear-base plates in endothelial cell basal medium-2 supplemented with 5% fetal bovine serum (FBS) and endothelial growth factors (EGM-2 MV bullet kit; Lonza, Walkersville, MD, USA) at 37 °C with 95% air, 5% CO_2_. After 3 days, non-adherent cells were removed and fresh medium was added and, subsequently, medium was changed every other day. At 7 days after sorting, cells became confluent and were used for experiments. Conditioned medium collected from the cell cultures was stored at − 80 °C for analysis of protein expression.

### Quantitative real time-PCR (qRT-PCR)

Total RNA was extracted from endothelial cells using the RNeasy Mini Kit (Qiagen, Valencia, CA, USA) with homogenization using a QIA shredder (Qiagen) according to the manufacturer’s instructions. The quality of total RNA samples was confirmed with a spectrophotometer (DeNovix DS-11; Scrum, Tokyo, Japan). Complementary DNA was generated from 1 μg RNA using high-capacity cDNA reverse transcription kits (Applied Biosystems, Foster City, CA, USA) according to the manufacturer’s instructions. qRT-PCR was performed with the THUNDERBIRD SYBR qPCR mix (TOYOBO, Tokyo, Japan) and Step One Plus (Applied Biosystems). Relative quantification of target gene transcript levels was standardized to those of the glyceraldehyde-3-phosphate dehydrogenase (GAPDH) gene and expressed as fold-changes derived from ΔΔCt values. Primers were from Thermo Fisher Scientific (Tokyo, Japan) and base sequences of primers are shown in Additional file 2: Table S1.

### Quantification of proteins

Proteins released from endothelial cells were quantified in samples of conditioned medium using ELISA kits for TGF-β1 (R&D Systems), platelet-derived growth factor (PDGF)-C (Cloud-Clone Corp, Wuhan, China) or connective tissue growth factor (CTGF) (Cusabio Life Science, Wuhan, China) according to the manufacturers’ protocols. Collagen content in conditioned medium was quantified using the Sircol Collagen Assay Kit (Biocolor Ltd., Carrickfergus, UK), according to the manufacturer’s protocol.

### Measurement of intracellular nitric oxide concentrations

Intracellular nitric oxide concentrations in endothelial cells were measured using 4- amino-5-methylamino-2′,7′-difluorofluorescein diacetate (DAF–FM/DA; Goryo Kayaku, Sapporo, Japan). Endothelial cells in each well were loaded with 6 μM DAF–FM/DA at 37 °C for 20 min in 200 μL modified Tyrode’s solution. After washing cells, a 200 μL aliquot of 1 μM thapsigargin (Sigma-Aldrich, St Louis, MO, USA) or vehicle in modified Tyrode’s solution was added. Five minutes later, DAF-FM fluorescence intensities of whole cells in each well were measured with excitation and emission wavelengths of 485 and 515 nm, respectively, using a fluorescence plate reader (Synergy H1; BioTek Instruments, Winooski, VT, USA). The fluorescence intensity ratios of thapsigargin-treated cells over those of vehicle-treated cells were calculated and compared for cells derived from both bleomycin and saline-treated mice.

### Measurement of prostaglandin I_2_ (PG-I_2_) production

PG-I_2_ released from endothelial cells was estimated by measuring the concentration of its stable metabolite, 6-keto PGF_1α_. After wash, 10 μM thapsigargin (Sigma-Aldrich) or vehicle was added in 200 μL modified Tyrode’s solution and cells were then incubated at 37 °C for 15 min. The concentrations of 6-keto PGF_1α_ in the medium were measured with an ELISA kit (Cayman Chemical Co., Ann Arbor, MI, USA) according to the manufacturer’s protocol. The concentration ratios of thapsigargin-treated over vehicle-treated cells were calculated and compared for cells derived from bleomycin-treated and saline-treated mice.

### Immunofluorescence staining

For immunofluorescence staining, cells were fixed in 4% paraformaldehyde for 10 min, incubated in blocking solution (5% goat serum and 0.5% Triton X-100 in PBS) for 30 min and incubated with unconjugated anti-α-SMA antibody (Sigma-Aldrich) and anti-CD31 antibody (Abcam, Cambridge, UK). They were then incubated with Alexa-Fluor 488 (green) conjugated anti-mouse IgG_2a_antibody (Invitrogen, Carlsbad, CA), Alexa-Fluor 568 (red) conjugated anti-rabbit IgG antibody (Abcam) and Hoechst 33,342 (Sigma-Aldrich) antibody for 60 min. Cells were imaged and images captured with IX83 (Olympus, Tokyo, Japan) and the ratios of α-SMA positive cells to the total cell numbers were calculated using Image J (National Institutes of Health, Bethesda, MD, USA).

### Ex vivo TGF-β1 stimulation

Experiments were also performed, where indicated, in cultured endothelial cells stimulated for 48 h with TGF-β1 (10 ng/mL, Peprotech, Rocky Hill, NJ).

### Statistical analysis

All data are presented as means ± standard error of the mean. Differences between two groups were evaluated with the Mann-Whitney’s U-test. For multiple group comparisons, Tukey’s test was performed. A value of *P* < 0.05 was considered to be statistically significant. All values were analyzed using JMP 9.0.0 (SAS Institute Japan, Tokyo, Japan).

## Results

### Intratracheal bleomycin administration induced damage and subsequent pulmonary fibrosis

**Fig. 1:**
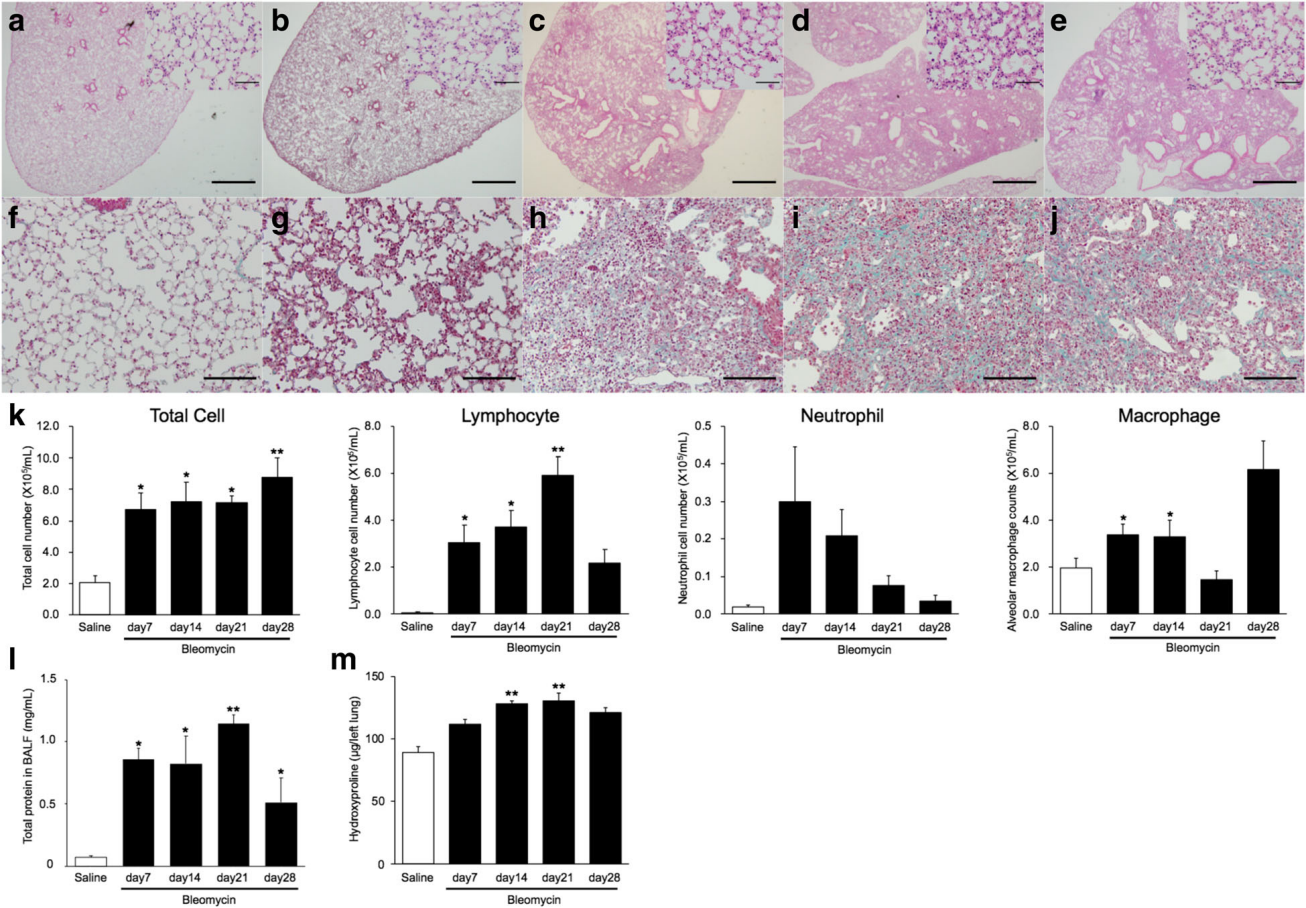
Intratracheal bleomycin-induced pulmonary fibrosis. Representative optical microscopy images of mouse lungs. Hematoxylin-eosin stain for saline (**a**) and bleomycin-treated lungs at day 7 (**b**), day 14 (**c**), day 21 (**d**) and day 28 (**e**). Scale bars indicate 500 μm (100 μm in inset). Masson’s trichrome stained saline (**f**) and bleomycin-treated lungs at day 7 (**g**), day 14 (**h**), day 21 (**i**) and day 28 (**j**). Scale bars indicate 200 μm. **k** Total and differential cell counts in bronchoalveolar lavage (BAL) fluid from saline and bleomycin-treated mice. BAL fluid was collected at days 7, 14, 21 and 28 after intratracheal bleomycin administration. Total cell numbers and lymphocyte counts in BAL fluid from bleomycin-treated lungs were significantly higher than in controls. Neutrophils in BAL fluid from bleomycin-treated lungs tended to be increased on day 7. **l** Total protein concentration in BAL fluid from saline and bleomycin-treated mice. BAL fluid was collected at days 7, 14, 21 and 28 after intratracheal bleomycin administration. Total protein concentrations in BAL fluid from bleomycin-treated lungs were significantly higher compared with that in controls. **m** Hydroxyproline content in lungs from saline and bleomycin-treated mice. Lung homogenates from saline and bleomycin-treated mice (*n* = 3 per group) were analyzed for hydroxyproline with a colorimetric assay. Hydroxyproline content was higher in the bleomycin-treated lungs. Results are means ± standard error, from three to seven mice. *p < 0.05, compared with saline-treated mice; **p < 0.05, compared with bleomycin-treated mice on day 7

Intratracheal administration of bleomycin to mice using a Microsprayer Aerosolizer led to a gradual weight decrease, with peak weight loss at about day 7, significantly lower than saline-treated mice. Gradually, the mice regained their weights. Pathological evaluation revealed patchy inflammatory cell infiltration and epithelial injury with reactive hyperplasia (Fig. 1a–e). Inflammatory cell infiltration in the alveolar walls was marked on day 7. Masson’s trichrome staining showed deposits of collagen fibers on day 14 and, most prominently, on day 21 in bleomycin-treated lungs (Fig. 1f–j). Total cell counts in the BAL were significantly elevated in bleomycin-treated mice and the number of lymphocytes peaked at day 21 (Fig. 1k). The concentration of total protein and TGF-β1 in BAL fluid was elevated over time following bleomycin administration, peaking on day 21 (Fig. 1l and Additional file 3: Fig. S2). The hydroxyproline content in the lung was increased over time after bleomycin administration (Fig. 1m). Bleomycin was reported to induce time-dependent cell injury, interstitial inflammation and deposition of extracellular matrix proteins [19]. The process is divided into two morphological phases, an inflammation predominant phase within one to two weeks after bleomycin administration and a fibrotic phase with a maximal response between the third and fourth week [19, 20]. Our findings indicated that inflammation was predominant on day 7 and fibrosis, with collagen deposition, peaked on day 21 in the bleomycin-treated lungs. We isolated endothelial cells on days 7 and 21 after bleomycin or saline administration, to represent cells from the inflammatory and fibrotic phases, respectively.

### Pulmonary endothelial cells were injured by bleomycin administration

As markers of endothelial injury, we examined expression of von Willebrand factor (vWF), plasminogen activator inhibitor-1 (PAI-1) and matrix metalloproteinase (MMP)-12 (Table 1). In endothelial cells from bleomycin-treated lungs, vWF and PAI-1 expression were elevated on day 7 and sustained on day 21, at values significantly higher than those from saline-treated lungs on the same days. MMP-12 expression was markedly upregulated in endothelial cells from bleomycin-treated lungs. These data suggested that endothelial cells were damaged even in the mouse pulmonary fibrosis model induced by intratracheal bleomycin.

**Table 1:** Relative mRNA expression associated with endothelial cell damage

	Day 7		Day 21	
	Saline	Bleomycin	Saline	Bleomycin
vWF	1.19 ± 0.25	3.09 ± 0.15^*^	1.33 ± 0.22	2.76 ± 0.14^*^
MMP-12	1.39 ± 0.20	18.0 ± 0.37^*^	0.87 ± 0.37	70.3 ± 0.52^*, **^
PAI-1	1.63 ± 0.31	6.05 ± 0.28^*^	1.72 ± 0.14	4.58 ± 0.32^*^

The table shows expression of endothelial damage markers, von Willebrand factor (vWF), matrix metalloproteinase (MMP)-12 and plasminogen activator inhibitor-1 (PAI-1) in lung endothelial cells isolated from saline-treated and bleomycin-treated mice. mRNA levels were determined by quantitative real time-PCR. Total RNA was isolated from magnetically sorted CD45^−^CD31^+^ mouse lung endothelial cells at 7 and 21 days after saline or bleomycin administration. PCR was performed using three or four independently prepared cDNA samples from endothelial cells. Results were normalized to expression levels in endothelial cells from untreated lungs at day 0 and are expressed as fold-changes. Data are means ± standard error, from three or four mice

^*^p < 0.05, compared with saline-treated mice on the same day

^**^p < 0.01, compared with bleomycin-treated mice on day 7

### Production of intracellular nitric oxide was attenuated in endothelial cells isolated from bleomycin-treated lungs

**Fig. 2:**
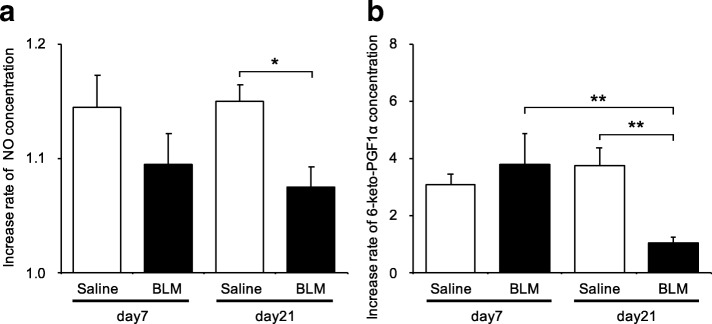
Functional performance of endothelial cells from bleomycin-treated lungs, assessed by response to thapsigargin. **a** Intracellular nitric oxide concentrations in endothelial cells were measured using DAF–FM/DA. Thapsigargin (1 μM) was added and the DAF–FM fluorescence intensity in whole cells was measured at 515 nm. The fluorescence intensity ratio indicates the relative fluorescence intensity of intracellular nitric oxide in thapsigargin-treated endothelial cells over that in untreated endothelial cells. The fluorescence intensity ratio of intracellular nitric oxide was significantly attenuated in endothelial cells from bleomycin-treated lungs at day 21, compared with in saline-treated lungs. **b** 6-Keto PGF_1α_ released from endothelial cells. The concentration of 6-keto PGF_1α_ in the culture medium was measured by ELISA after incubation of endothelial cells with 10 μM thapsigargin. The relative concentration ratio indicates the concentration of 6-keto PGF_1α_ in thapsigargin-treated endothelial cells over that in untreated cells and these ratios were compared for cells from saline and bleomycin-treated mice. Relative rates of 6-keto PGF_1α_ production were significantly attenuated in thapsigargin-stimulated endothelial cells from bleomycin-treated lungs, compared with in those from saline-treated lungs, both isolated on day 21. These levels were also attenuated relative to those in endothelial cells from bleomycin-treated lungs that were not stimulated by thapsigargin at the same day. Data are means ± standard error from three or four mice. *p < 0.05, **p < 0.01

To investigate properties of endothelial cells isolated from bleomycin-treated mouse lungs, we examined intracellular nitric oxide production in response to thapsigargin stimulation. Thapsigargin is a selective inhibitor of the endoplasmic reticulum (ER) Ca^2+^-ATPase and increases intracellular Ca^2+^ levels through plasma membrane store-operated Ca^2+^ channels that are activated by depletion of ER Ca^2+^ stores. Thapsigargin stimulation is believed to lead to production of nitric oxide and PG-I_2_. Intracellular nitric oxide concentrations were significantly elevated by 1 μM thapsigargin stimulation in endothelial cells from either bleomycin or saline-treated mice. However, nitric oxide levels were significantly lower in endothelial cells from bleomycin-treated mice on day 21 (Fig. 2a, *p* = 0.0148).

### PG-I_2_ release was attenuated in endothelial cells isolated from bleomycin-treated lungs

Because of the rapid metabolism of PG-I_2_, its concentration was estimated based on that of its stable metabolite, 6-keto PGF_1α_. To investigate PG-I_2_ release from endothelial cells in response to bleomycin, we therefore measured changes in 6-keto PGF_1α_ concentrations in the culture medium of cells stimulated with thapsigargin. Thapsigargin simulation increased 6-keto PGF_1α_ production in both endothelial cells from both bleomycin-treated and saline-treated mice. In the cells isolated on day 7 from bleomycin-treated mice, the thapsigargin-induced increase in 6-keto PGF_1α_ was similar to that in cells from saline-treated animals. However, the response to thapsigargin was significantly lower in cells isolated on day 21 from bleomycin-treated mice (Fig. 2b, *p* = 0.0054). This suggested that endothelial cell function, assessed by thapsigargin reactivity, was attenuated in endothelial cells at the fibrotic phase.

### Fibrotic mediators and NOSs in endothelial cells isolated from bleomycin-treated lungs

**Fig. 3:**
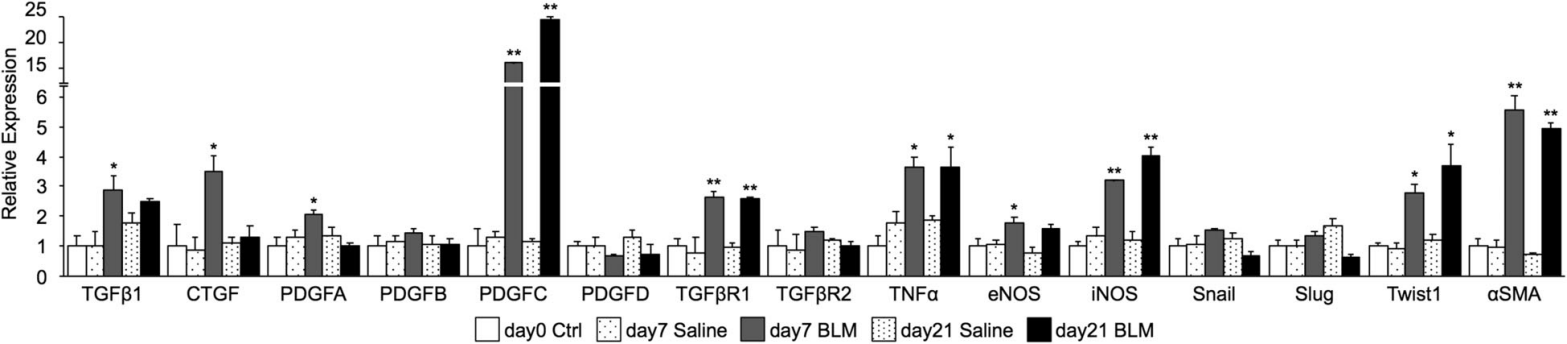
Expression of mediators, determined by quantitative real-time PCR. Levels of mRNA for various mediators were compared in endothelial cells from saline and bleomycin-treated mouse lungs. Quantitative real-time PCR was performed using three or four independently prepared cDNA samples from endothelial cells harvested from saline or bleomycin-treated lungs on days 7 and 21. Gene expression was calculated asΔCt, the Ct of a gene of interest minus the Ct of GAPDH from the same sample. Results were normalized to expression levels in endothelial cells from untreated lungs at day 0 and are means from three experiments. Data are means ± standard error of the mean for three or four mice. *p < 0.05, **p < 0.01, compared with saline-treated mice

**Fig. 4:**
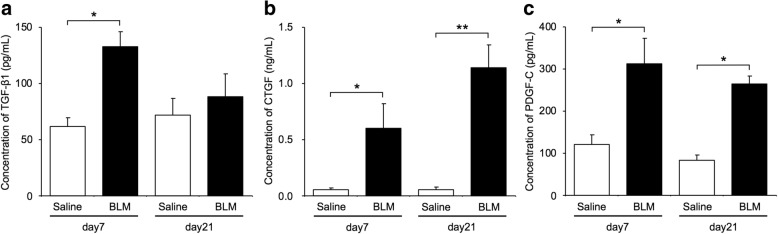
Fibrotic mediator proteins released from endothelial cells. Protein levels of TGF-β1 (**a**), CTGF (**b**) and PDGF-C (**c**) were quantified by ELISA. Concentrations of TGF-β1, CTGF and PDGF-C were significantly higher in the culture medium of endothelial cells from bleomycin-treated lungs, compared with those from saline-treated lungs. Data are means ± standard error of the mean for three to six mice. *p < 0.05, **p < 0.01, compared with saline-treated mice

**Fig. 5:**
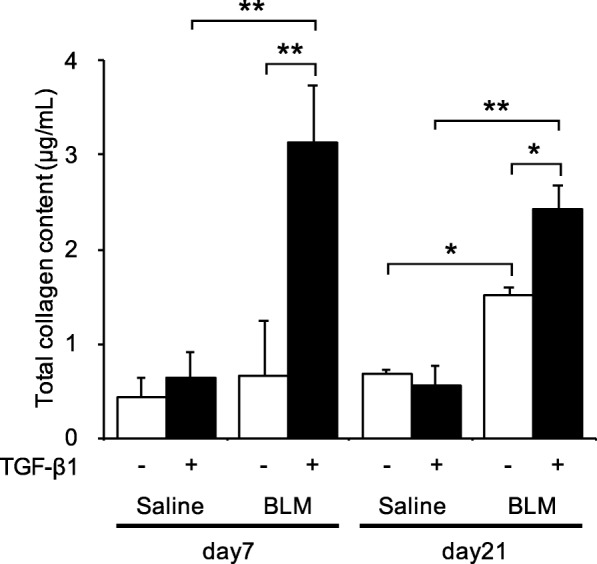
Total collagen content in endothelial cells, with or without TGF-β1. Total soluble collagen content was measured by the Sircol assay. The collagen content in the culture medium from endothelial cells isolated from bleomycin-treated lungs was higher than samples from saline-treated lungs. TGF-β induced significantly increased collagen content in endothelial cells from bleomycin-treated lungs. However, in medium from endothelial cells isolated from saline-treated mice lungs, TGF-β did not affect total collagen content. Results are means ± standard error of the mean from three or four mice per group. *p < 0.05, **p < 0.01, compared with saline-treated mice

mRNA expression of fibrotic mediators and NOSs was evaluated (Fig. 3). Levels of TGF-β1 mRNA were significantly elevated on day 7 compared with those in endothelial cells from saline-treated mice. On day 21, there was no significant difference in expression between endothelial cells from bleomycin and saline-treated mice. Expression of CTGF was increased in cells isolated at day 7 after bleomycin administration. Among PDGF family members, PDGF-C expression was elevated in endothelial cells from bleomycin-treated mouse lungs on days 7 and 21. Protein levels of TGF-β1, PDGF-C and CTGF released from endothelial cells from bleomycin-treated mice were higher than those in cells from saline-treated mice (Fig. 4). iNOS expression was elevated in endothelial cells from bleomycin-treated mouse lungs at days 7 and 21. eNOS levels were elevated in cells only from day 7. The amount of collagen released into the culture medium of cells from bleomycin-treated lungs at day 21 was significantly higher than in other endothelial preparations (Fig. 5).

### Addition of TGF-β to endothelial cells isolated from bleomycin-treated lungs

**Fig. 6:**
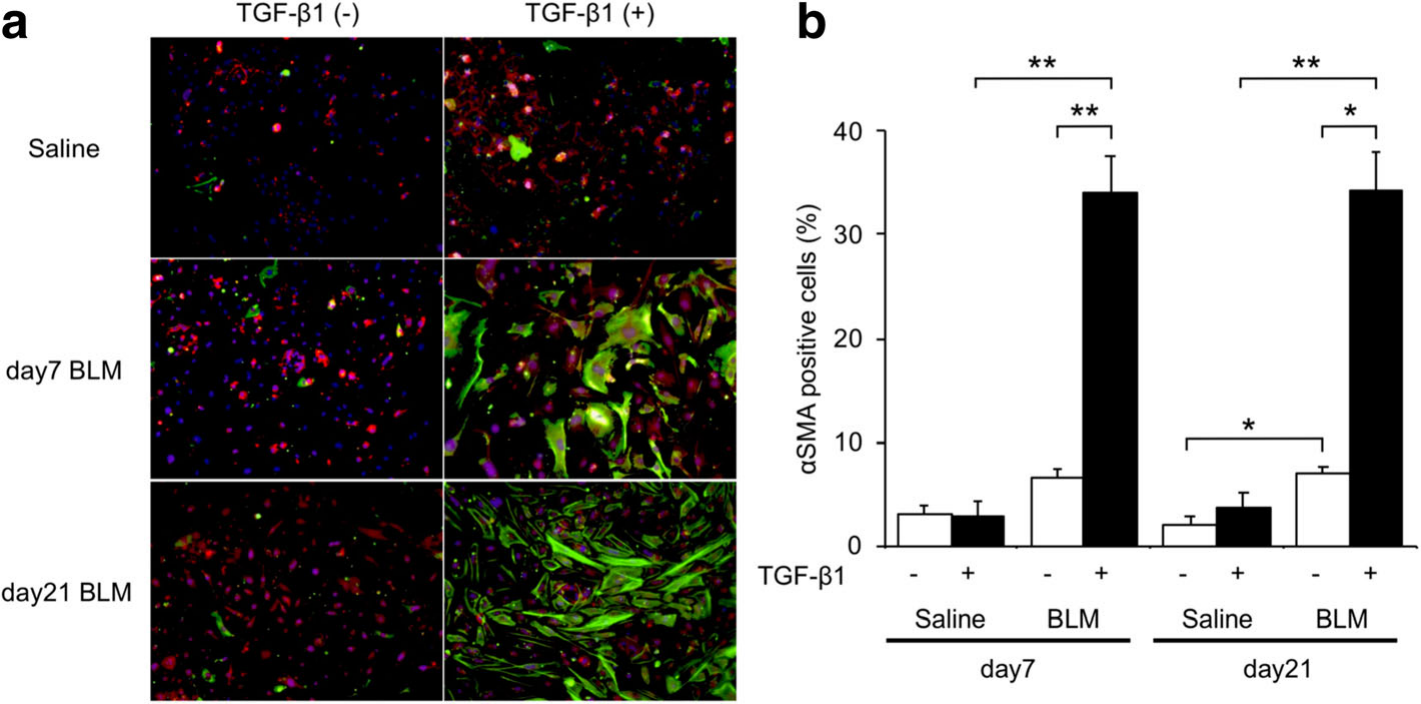
TGF-β1-induced increased α-SMA staining in endothelial cells from bleomycin-treated lungs. **a** Shown are representative fluorescence microscopy images of endothelial cells from bleomycin-treated lungs, cultured with or without TGF-β1. The α-SMA (green) and CD31 (red) stained images were merged with those showing a nuclear stain (Hoechst, blue). **b** Comparison of the percentage of α-SMA positive endothelial cells from saline and bleomycin-treated lungs, cultured with or without TGF-β1. Cells were imaged and images captured with IX83 and the percentage of α-SMA positive cells expressed as the percentage of total cell number, estimated from Hoechst 33,342 nuclear staining. The percentage of α-SMA positive cells was significantly elevated in cells from bleomycin-treated lungs and in any endothelial cells treated with TGF-β1, compared with in the corresponding controls. TGF-β1 treatment further enhanced the percent of α-SMA stained cells in lung endothelial cultures from bleomycin-treated mice. Data are means ± standard error, from three to four mice. * *p* < 0.05, ** *p* < 0.01

**Fig. 7:**
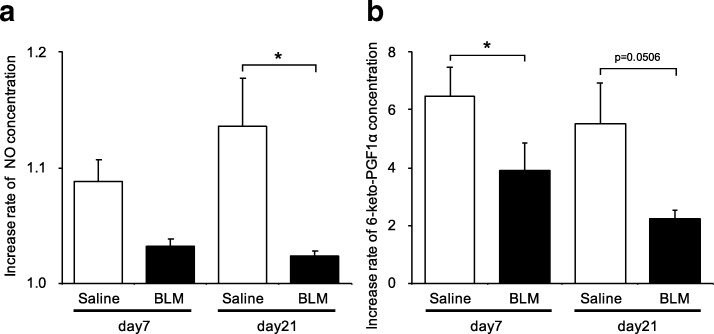
Effects of TGF-β on the functional properties of lung endothelial cells isolated from bleomycin-treated mice. **a** Intracellular nitric oxide response to TGF-β was significantly attenuated in cells isolated on day 21 after bleomycin treatment (*p* = 0.0138, compared with cells from saline-treated mice). **b** The increase in 6-keto-PGF_1α_ levels caused by TGF-β was attenuated in cells isolated on day 7 or 21 from bleomycin-treated mice (*p* = 0.0206, *p* = 0.0506, respectively, compared with cells from saline-treated mice). Data are means ± standard error, from three to four mice. *, *p* < 0.05

To further explore the functional and phenotypic changes of endothelial cells in bleomycin-treated lungs, we performed additional studies in which TGF-β was added to endothelial cells. The collagen content from lung endothelial cells treated with TGF-β was significantly higher than in cells without TGF-β treatment (Fig. 5). In endothelial cells from saline-treated mouse lungs, adding TGF-β did not increase collagen content. Next, we fluorescently stained endothelial cells for α-SMA and CD31 and observed them with a fluorescence microscope (Fig. 6). Even without TGF-β1, endothelial cells from bleomycin-treated mouse lungs had increased α-SMA staining. The percentage of α-SMA positive cells on day 21 was 7.0%, significantly higher than in cells from saline-treated mouse lungs on the same day (2.1%, *p* < 0.05). When stimulated with TGF-β1, approximately one third of bleomycin-treated lung endothelial cells had elevated α-SMA staining. The percentage of cells with positive staining was significantly higher in cells from bleomycin-treated than from vehicle-treated lungs (Fig. 6, *p* < 0.01). These changes in α-SMA levels were confirmed at the mRNA level (Table 2 and Fig. 3). Interestingly, TGF-β did not increase the percentage of α-SMA-positive cells in lung endothelial cells from saline-treated mice. Regarding the function of the endothelial cells, TGF-β addition to endothelial cells from day 21 after bleomycin treatment decreased their intracellular nitric oxide concentrations to values lower than in the same cells without TGF-β (Fig. 7a). Similarly, the thapsigargin-induced increase in 6-keto PGF_1α_ in endothelial cells from bleomycin-treated mice was attenuated by TGF-β (Fig. 7b). We also evaluated mRNA expression of PDGF-C, CTGF, iNOS, eNOS and Twist-1 in cells after TGF-β treatment (Table 2). After TGF-β treatment, PDGF-C expression in endothelial cells was increased and that of iNOS and eNOS was decreased. Expression of Twist-1 in lung endothelial cells, already increased if they were isolated after bleomycin treatment, was further increased by TGF-β.

**Table 2:** Relative mRNA expression in endothelial cells, with and without TGF-β1

	Day 7		Day 21	
TGF-β1	–	+	–	+
CTGF	3.49 ± 0.54	4.84 ± 0.10	1.27 ± 0.41	2.61 ± 0.14
PDGF-C	16.0 ± 0.07	22.8 ± 0.29	24.4 ± 0.37	79.5 ± 0.56^*^
eNOS	1.75 ± 0.21	0.16 ± 0.42^**^	1.59 ± 0.11	0.07 ± 0.71^**^
iNOS	3.20 ± 0.02	0.24 ± 0.32^**^	4.05 ± 0.28	0.11 ± 0.75^**^
Twist-1	2.76 ± 0.31	7.95 ± 0.23^**^	3.71 ± 0.72	14.3 ± 0.59^*^
α-SMA	5.60 ± 0.80	18.6 ± 0.16^*^	4.97 ± 0.29	35.8 ± 0.29^*^

The table shows gene expression of connective tissue growth factor (CTGF), platelet-derived growth factor (PDGF)-C, inducible nitric oxide synthase (iNOS), endothelial nitric oxide synthase (eNOS), Twist-1, and α-smooth muscle actin (α-SMA) in lung endothelial cells from bleomycin-treated mice, with and without TGF-β1 (10 mg/mL) treatment. mRNA levels were determined by quantitative real time-PCR. Total RNA was isolated from endothelial cells at 7 and 21 days after bleomycin administration. PCR was performed using three to four independently prepared cDNA samples from endothelial cells. Results were normalized to expression levels in endothelial cells from untreated lungs at day 0 and are expressed as fold-changes. Data are means ± standard error of the mean, from three or four mice

^*^p < 0.05 versus cells without TGF-β1

^**^p < 0.01 versus cells without TGF-β1 on day 21 and with TGF-β1 on day 7

## Discussion

In this study, we isolated endothelial cells from lung tissues with bleomycin-induced fibrosis and investigated the functional changes occurring during fibrogenesis. Levels of vWF, MMP-12 and PAI-1 were elevated, indicating continuous endothelium injury, on day 7 and as late as day 21. Expression of mediators related to fibrosis and collagen production was also increased. PG-I_2_ and nitric oxide production in response to thapsigargin, representing intrinsic functions of endothelium, were sustained in endothelial cells harvested at 7 days after bleomycin administration but were lower in those isolated at 21 days. In endothelial cells stimulated with TGF-β, α-SMA expression and collagen production were increased in cells from bleomycin-treated, compared with vehicle-treated, mouse lungs. TGF-β treatment also decreased thapsigargin-induced PG-I_2_ and nitric oxide production by the cells. Bleomycin exposure induced functional changes in lung endothelial cells, potentially related to their involvement in fibrogenesis.

Various mediators, including tumor necrosis factor-α (TNF-α) [24], CTGF [25], interleukin (IL)-18 and IL-1β [26], are believed to mediate bleomycin-induced pulmonary inflammation and fibrosis. These mediators are expressed primarily in alveolar macrophages, epithelial cells, fibroblasts and immune cells. In the lungs, TGF-β, the major fibrotic cytokine, is produced by a wide variety of cell types, including alveolar macrophages, neutrophils, activated alveolar epithelial cells, fibroblasts and myofibroblasts [18, 27–31]. Zhang et al. showed that fibroblasts, myofibroblasts and eosinophils strongly express TGF-β mRNA and protein in endotracheal cells from rats with bleomycin-induced pulmonary fibrosis [32]. However, their study did not focus on endothelial cells. Yan et al. purified endothelial cells from rats using a tissue explant method of bleomycin-induced fibrosis and identified endothelial cells by vWF immunofluorescence and cell morphology. Endothelial cells from the bleomycin-treated rats showed increased secretion of CTGF from days 7 to 28 [33]. Leach et al. administered bleomycin subcutaneously and then purified endothelial cells from the treated animals by cell sorting using flow cytometry [21]. In endothelial cells harvested on the fourth week after bleomycin administration, there was overexpression of the fibrotic mediators, CTGF, PAI-1 and osteopontin. These investigators proposed that, because endothelial injury markers were elevated between the first and second week, endothelial cell injury preceded development of fibrosis. In our study, gene expression of the fibrotic mediators, CTGF and TGF-β, was elevated in endothelial cells at 7 days after bleomycin administration. In addition, we confirmed that bleomycin treatment increased expression of these mediators at the protein level. There were differences in the timing of endothelial injury and fibrotic markers elevations. In our study, levels of endothelium injury markers, vWF, MMP-12 and PAI-1, and fibrotic mediators were elevated on day 7 and sustained on day 21. In the study of Leach et al., levels of endothelium injury markers were elevated on day 28 after bleomycin administration and there was no difference in the expression of TGF-β1 mRNA between endothelial cells from bleomycin-treated and saline-treated lungs. These differences might be explained by the bleomycin administration route used. Our intratracheal administration might have induced massive and early damage, compared with the subcutaneous route. Although the timing of these elevations was not consistent, our study and that of Leach et al. confirmed production of fibrotic mediators by endothelial cells from bleomycin-induced fibrotic lungs. Because these mediators directly recruit and activate fibroblasts to produce collagen, endothelial cells may contribute to the development of tissue fibrosis by producing such agents in response to bleomycin. Leach et al. also emphasized the contribution of endothelial cells to macrophage recruitment in inflammation-driven fibrosis and showed the up-regulation of C-C motif chemokine ligands and inflammatory mediators. We tested TNF-α expression as an inflammation related factor. In our model, the expression of TNF-α was elevated in endothelial cells at 7 days and sustained on day 21 after bleomycin administration, which indicates that inflammation was connected with our bleomycin-induced fibrosis.

As other fibrotic mediators, we examined expression of four PDGF receptor ligands. There were no significant differences in PDGF-A, B and D mRNA levels between endothelial cells from bleomycin-treated and saline-treated mouse lungs. In contrast, PDGF-C expression was elevated in endothelial cells from bleomycin-treated mouse lungs. TGF–β further enhanced PDGF-C expression. PDGF is a potent mitogen for fibroblasts. Its overproduction can induce heart, liver and renal fibrosis. In the lung, PDGF-C expression was elevated in the bleomycin model, localized to patchy areas of lung fibrosis [34]. Various cell types, including epithelial, mesenchymal and inflammatory cells, were reported to produce PDGF-C, contributing to fibrosis [35]. In this study, we showed that PDGF-C expression was also elevated in endothelial cells during bleomycin-induced pulmonary fibrosis.

In addition to the production of fibrotic mediators, endothelial cells play critical roles in several physiological and pathological processes [12] through the activities of endothelium-derived relaxing and/or constricting factors. Two endothelium-derived relaxing factors, nitric oxide and PG-I_2_, are constitutively released by endothelial cells and their synthesis is increased in response to agonists [12]. Both agents have multiple effects, modulating platelet aggregation, inhibiting leukocyte adhesion and controlling vascular smooth muscle cell proliferation [36]. We examined production of nitric oxide and PG-I_2_ in endothelial cells from bleomycin-induced fibrotic lung tissues. Although these cells produced nitric oxide and PG-I_2_ when stimulated with thapsigargin, the response in endothelial cells from fibrotic lungs was significantly weaker than in cells from normal lungs. Conversely, mRNA expression of iNOS was elevated, relative to the normal cells, in endothelial cells from bleomycin-induced fibrotic lungs. Because iNOS expression is stimulated by inflammation, increased expression of its transcript would be expected to occur after bleomycin administration. Almudever et al. showed that levels of tetrahydrobiopterin (BH4), a cofactor of NOS, were decreased in IPF, an effect that would lead to uncoupling of NOS activity [37]. Although BH4 levels were not examined in our experiments, it is possible that iNOS was uncoupled in the bleomycin-treated mouse lungs, leading to less nitric oxide formation and more oxidative stress. The weak nitric oxide response may have been related to the finding that eNOS knockout animals had prolonged fibrosis, compared with wildtypes, after bleomycin exposure [13].

In our study, we added TGF-β to endothelial cells to determine whether it would induce additional changes in endothelial function. Regarding nitric oxide and PG-I_2_ production, interestingly, TGF-β further suppressed the weak nitric oxide and PG-I_2_ production by endothelial cells from bleomycin-treated mouse lungs. However, TGF-β did not affect levels of these mediators produced by cells from saline-treated control mice. Finder et al. showed that TGF-β caused decreased nitric oxide production by rat pulmonary artery smooth muscle cells [38]. Our data indicated that inhibition of nitric oxide production by TGF-β also occurred in pulmonary endothelial cells. Interestingly, TGF-β enhanced the number of α-SMA-positive cells and collagen production by endothelial cells from bleomycin-treated mouse lungs. TGF-β stimulates type I collagen transcription, promotes synthesis of extracellular matrix components and is a central regulator of recruitment, activation and differentiation of myofibroblasts at the early stages of tissue repair [16, 27] and is highly involved in development of organ fibrosis [6]. We speculated that bleomycin pretreatment caused endothelial cells to be susceptible to further stimulation, by for example, TGF-β, as in our study. In our study, endothelial cells were substantially changed, functionally (collagen production) as well as phenotypically (transformed into α-SMA positive cells) by both bleomycin exposure and TGF-β. In general, patients with pre-existing ILD are known to be at risk for acute exacerbation of ILD [39]. It is conceivable that bleomycin-induced fibrosis represents a priming state that is highly sensitive to any kind of stimulation and can then be readily driven to exacerbated fibrosis.

Another mechanism for endothelial cell participation in the pathogenesis of fibrosis, may involve secretion and deposition of excess collagen in tissues through the endothelial–mesenchymal transition. During the endothelial–mesenchymal transition, endothelial cells lose their endothelial phenotype, such as expression of CD31 and vascular endothelial cadherin, and acquire a fibroblast-like mesenchymal phenotype, expressing α-SMA, vimentin and type I collagen. Although several studies demonstrated that a partial epithelial–mesenchymal transition participated in pathologic fibrogenesis [40], there has been insufficient investigation of the role of myofibroblasts from the endothelial–mesenchymal transition in pulmonary fibrosis. Hashimoto et al. showed that areas of fibrotic involvement contained significant numbers of myofibroblasts originating from endothelial cells in bleomycin induced pulmonary fibrosis [41]. TGF-β and activated Ras induced de novo α-SMA expression in microvascular endothelial cells. We observed elevated expression of α-SMA and Twist-1, a transcription factor involved in endothelial–mesenchymal transition [42], in endothelial cells from bleomycin-treated mouse lungs, indicating existence of an endothelial–mesenchymal transformation. Furthermore, adding TGF-β significantly enhanced α-SMA and Twist-1 expression in such cells. TGF-β induced mesenchymal transformation may be prominent in existing fibrosis. These findings, together, supported the conclusion that endothelial cells may adopt a fibroblast-like phenotype, through an endothelial–mesenchymal transformation, during fibrosis. In the present study, the connections between the nitric oxide pathway and this endothelial–mesenchymal transition remain unclear. Further studies are warranted to clarify the detailed mechanistic relationship between them.

## Conclusions

Endothelial cells from lungs subjected to bleomycin-induced fibrosis were functionally altered and had a phenotype similar to that of myofibroblasts, potentially contributing to progression of pulmonary fibrosis. Pulmonary fibrosis is a lethal disease requiring elucidation of its mechanisms and, thereby, development of new therapies. A pathway involving endothelial cells may be a new therapeutic target for pulmonary fibrosis.

## Additional files

Additional file 1: **Figure S1.** Purity of magnetically sorted mouse lung CD45^−^CD31^+^ cells. The purity was confirmed using flow cytometry with antibodies to CD31 and CD45. Representative example of dot plots obtained from magnetically sorted mouse lung CD45^−^CD31^+^ cells. Figures indicate percentages of cells expressing CD45 and CD31. The purity was > 90% in the three experiments. (PDF 124 kb)

Additional file 2: **Table S1.** Primers used for quantitative real-time PCR. (DOCX 21 kb)

Additional file 3: **Figure S2.** TGF-β1 concentrations in BAL fluid after administration of bleomycin. TGF-β1 concentrations in BAL fluid were gradually increased, with a significant increase on day 21 after bleomycin treatment (*p* = 0.0025 and *p* = 0.0235, compared with saline-treated mice and bleomycin-treated mice on day 7, respectively). (PDF 21 kb)

## Abbreviations

BALF: Bronchoalveolar lavage fluid
BH4: Tetrahydrobiopterin
BLM: Bleomycin
CTGF: Connective tissue growth factor
DAF-FM/DA: 4-amino-5-methylamino-2′,7′-difluorofluorescein diacetate
eNOS: Endothelial nitric oxide synthase
IL: Interleukin
ILD: Interstitial lung disease
iNOS: Inducible nitric oxide synthase
IPF: Idiopathic pulmonary fibrosis
MMP-12: Matrix metalloproteinase
PAI-1: Plasminogen activator inhibitor-1
PDGF: Platelet-derived growth factor
PG-I_2_: Prostaglandin I_2_
TGF-β: Transforming growth factor-β
TNF-α: Tumor necrosis factor-α
UIP: Usual interstitial pneumonia
vWF: von Willebrand factor
α-SMA: α-smooth muscle actin
