# Supplementary figures and images for: Changes in pulmonary endothelial cell properties during bleomycin-induced pulmonary fibrosis
Source: Respir Res. 2018 Jun 26;19:127. doi: 10.1186/s12931-018-0831-y (PMC6019800; doi:10.1186/s12931-018-0831-y)

**Unstained**

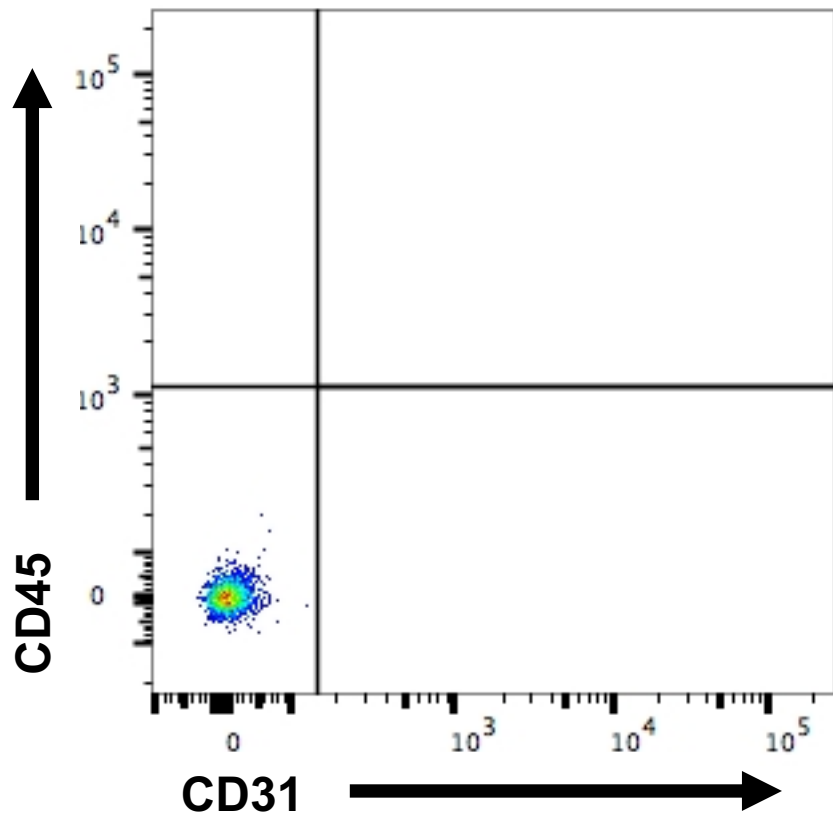

**Stained**

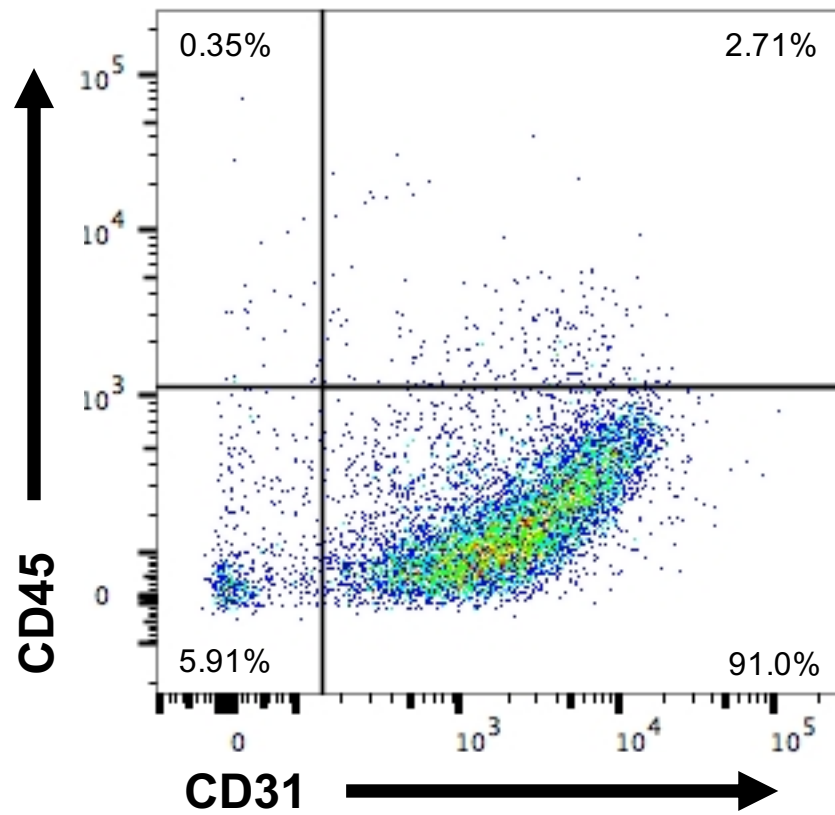

Supplement: Supplementary file 1 — Figure S1. Purity of magnetically sorted mouse lung CD45−CD31+ cells. The purity was confirmed using flow cytometry with antibodies to CD31 and CD45. Representative example of dot plots obtained from magnetically sorted mouse lung CD45−CD31+ cells. Figures indicate percentages of cells expressing CD45 and CD31. The purity was > 90% in the three experiments. (PDF 124 kb) [file 12931_2018_831_MOESM1_ESM.pdf]

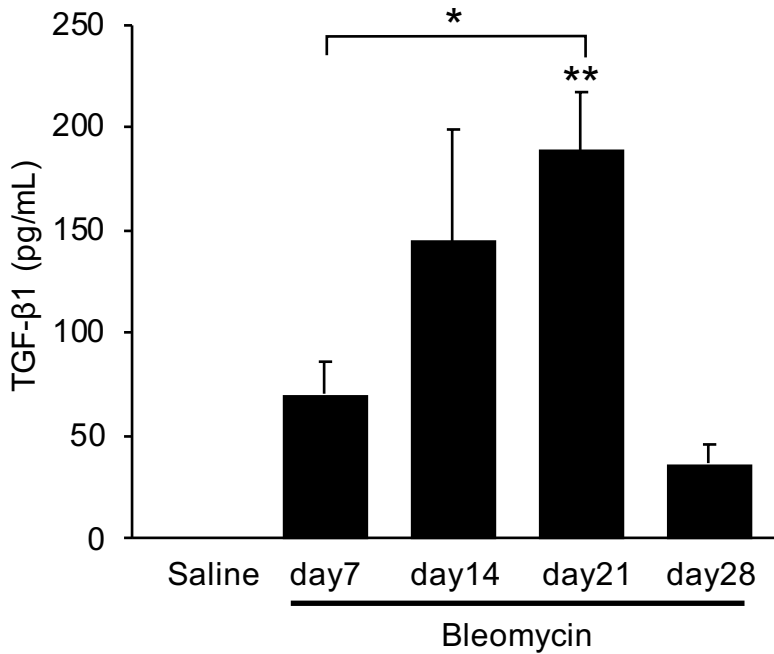

Supplement: Supplementary file 3 — Figure S2. TGF-β1 concentrations in BAL fluid after administration of bleomycin. TGF-β1 concentrations in BAL fluid were gradually increased, with a significant increase on day 21 after bleomycin treatment (p = 0.0025 and p = 0.0235, compared with saline-treated mice and bleomycin-treated mice on day 7, respectively). (PDF 21 kb) [file 12931_2018_831_MOESM3_ESM.pdf]
