# Supplementary material for: Changes in pulmonary endothelial cell properties during bleomycin-induced pulmonary fibrosis
Source: Respir Res. 2018 Jun 26;19:127. doi: 10.1186/s12931-018-0831-y (PMC6019800; doi:10.1186/s12931-018-0831-y)
Supplement: Supplementary file 2 — Table S1. Primers used for quantitative real-time PCR. (DOCX 21 kb) [file 12931_2018_831_MOESM2_ESM.docx]

Additional file 3: Table S1. Primers used for quantitative real-time PCR

|  | Forward | Reverse |
| --- | --- | --- |
| vWF | TTGGGAACTCCTGGAAAGTG | GATGTTGTTGTGGCAAGTGG |
| MMP-12 | TGATGCAGCTGTCTTTGACC | TGGGAAGTGTGTGGAAATCA |
| PAI-1 | AGGATCGAGGTAAACGAGAGC | GCGGGCTGAGATGACAAA |
| TGF-β1 | TGGAGCAACATGTGGAACTC | CAGCAGCCGGTTACCAAG |
| CTGF | GAGTGTGCACTGCCAAAGAT | GGCAAGTGCATTGGTATTTG |
| PDGF-A | GGAACTGAACAGGTGGGAGA | ATTCCACGTAAGGCCATCAG |
| PDGF-B | ATGTGCCCTTCAGTCTGCTC | GAGACAGGTCTCCTGCCCTA |
| PDGF-C | GCCCGAAGTTTCCTCATACA | ACACTTCCATCACTGGGCTC |
| PDGF-D | CGAGGGACTGTGCAGTAGAAA | TTGATGGATGCTCTCTGCGG |
| eNOS | TCCGGAAGGCGTTTGATC | GCCAAATGTGCTGGTCACC |
| iNOS | CACCTTGGAGTTCACCCAGT | ACCACTCGTACTTGGGATGC |
| TNF-α | CCCAGACCCTCACACTCAGATC | TGCTCCTCCACTTGGTGGTT |
| TGF-βR1 | CAGAGGGCACCACCTTAAAA | CTCGCCAAACTTCTCCAAAC |
| TGF-βR2 | ATGCATCCATCCACCTAAGC | TGTCGCAAGTGGACAGTCTC |
| Twist-1 | CGCACGCAGTCGCTGAACG | GACGCGGACATGGACCAGG |
| Snail | CCACTGCAACCGTGCTTTT | GTGCTTGTGGAGCAAGGAC |
| Slug | TACAGCCCCATCACTGTGTGGAC | CGCCCCAAAGATGAGGAGTATCC |
| α-SMA | TGTGCTGGACTCTGGAGATG | GAAGGAATAGCCACGCTCAG |
| GAPDH | TGTGTCCGTCGTGGATCTGA | CCTGCTTCACCACCTTCTTGA |

vWF, von Willebrand factor; MMP-12, matrix metalloproteinase; PAI-1, plasminogen activator inhibitor-1; TGF-β, transforming growth factor-β; TGF-βR, transforming growth factor-β receptor; CTGF, connective tissue growth factor; PDGF, platelet-derived growth factor; iNOS, inducible nitric oxide synthase; eNOS, endothelial nitric oxide synthase; TNF-α, tumor necrosis factor-α; TGF-βR1, TGF-β receptor type I; TGF-βR2, TGF-β receptor type II; α-SMA, α-smooth muscle actin; GAPDH, glyceraldehyde-3-phosphate dehydrogenase. Sequences are shown as (left to right) 5' to 3'.
